# Moderating role of age in the relationship between ingroup range and intention to help during the COVID-19 pandemic

**DOI:** 10.1371/journal.pone.0316316

**Published:** 2025-01-24

**Authors:** Inyeong Lee, Hyekyung Park

**Affiliations:** 1 Department of Psychology, Sogang University, Seoul, South Korea; 2 Department of Psychology, Sungshin Women’s University, Seongbuk-gu, Seoul, Republic of Korea; Guizhou University, PAKISTAN

## Abstract

This study explored the relationship between the ingroup range of individuals and their willingness to assist various social groups during the COVID-19 pandemic and whether or not age moderates this relationship. A total of 291 South Koreans (*M*_*age*_ = 31.91 years, *SD* = 11.99) participated in an online survey and provided data on ingroup range, intention to provide help, and age. The results demonstrated that individuals with a broader ingroup range displayed a stronger willingness to distribute quarantine supplies for COVID-19 across diverse social groups (*β* = .12, *p* < .05). This finding aligned with the ingroup bias phenomenon, in which individuals tend to favor one’s group. Importantly, age moderated the association between ingroup range and willingness to help (*B* = −.19, *p* < .05). Specifically, the intention of younger individuals (*θ*_X → Y_|(*M* = −11.985) = 4.40, *CI* = 1.79–7.01) and middle-aged individuals (*θ*_X → Y_|(*M* = .000) = 2.14, *CI* = .31–3.98) to assist diverse social groups significantly varied according to their ingroup range, whereas no such relationship existed among older individuals (*θ*_*X → Y*_|(*M* = 11.985) = −.11, *CI* = −2.64–2.42). We provided an in-depth discussion into the potential reasons that underlie the stronger willingness of older individuals to extend aid beyond their ingroup range during the pandemic compared with those of younger individuals. We then suggested strategies for encouraging acts of prosociality during crises, such as the COVID-19 pandemic, which particularly emphasize the pattern observed in younger participants with a narrow ingroup range.

## Introduction

The COVID-19 pandemic, which began in late 2019, had changed daily life. Mask wearing indoors and frequent hand washing had become the norm; work, education, and other activities, which were previously conducted face-to-face, had become virtual. On May 5, 2023, the World Health Organization (WHO) lifted the declaration of COVID-19 as a public health emergency of international concern. Although most countries have now moved toward pre-pandemic routines, at least some of the lifestyle changes due to COVID-19, such as wearing masks in public and the spread of virtual meetings, have persisted beyond the pandemic.

The COVID-19 pandemic had not only changed lifestyles. Fear of the pandemic had caused people to prioritize their personal or group interests over the public good. For example, major concerns had previously emerged about vaccine imbalance due to the monopolization of vaccines by a few wealthy countries. Shortly after the dissemination of COVID-19 vaccines, vaccination rates in high-income countries had exceeded 70%, whereas less than 10% of the population in low-income countries had been vaccinated as of January 9, 2022 [1]. Just before the pandemic ended, with vaccine supplies more stable than before, low-income countries had continued to be less than 30% vaccinated as of February 27, 2023 [1]. In another example, panic buying had become a problem during the COVID-19 pandemic with people stockpiling not only items that were essential for quarantine (e.g., face masks) but also everyday necessities (e.g., toilet paper and cleaning supplies).

This tendency to prioritize one’s interests and those of one’s group, which had become prominent during the pandemic, rendered mitigating the spread of COVID-19 increasingly difficult and made oneself vulnerable to infection as a result. Prioritizing the common good over individual interest was important to end the COVID-19 pandemic. Therefore, being prosocial, that is, giving to those in need, may be one of the most effective strategies for overcoming any future major health crisis facing humanity. The present study examines the factors that predict individuals’ intention to engage in prosocial behavior during the COVID-19 pandemic. In particular, we focus on ingroup bias by examining whether or not the perceived ingroup range of an individual is related to the intention to help different social groups. Moreover, we test whether or not age moderates the relationship between ingroup range and intention to help. This study is of importance, because ingroup bias is a relatively universal phenomenon observed in various societies [2,3], although the extent of bias may vary according to the characteristics of a society. This study intends to provide a foundation for addressing inequality in resource distribution in the face of the COVID-19 pandemic as well as future outbreaks of infectious diseases.

### Ingroup bias during the COVID-19 pandemic

Numerous studies on intergroup behavior have consistently demonstrated that individuals tend to offer more assistance and distribute more resources to members of their group compared with those outside of it [4–6]. This propensity extends to offering greater help to ingroup members following critical situations that require intervention [7], natural disasters [8,9], and even disasters precipitated by human error [10]. This prosocial bias toward the ingroup, which is termed *ingroup bias*, is observed not only among adults but also in young children [6,11].

Ingroup bias was notably present during the COVID-19 pandemic. For instance, a study involving British participants demonstrated increased willingness to donate to their group (British) over an outgroup (Chinese) suffering from COVID-19. This inclination was particularly pronounced among those who strongly identified as British [12]. Beyond the matter of resource allocation, research indicated that a heightened perception of vulnerability to infectious diseases is linked to more negative attitudes toward outgroups [13] and an increase in ethnocentric attitudes [14]. In essence, perceiving oneself as susceptible to a contagious disease seemingly fostered an inclination to favor one’s group while excluding others.

Why then do these phenomena occur? From an evolutionary psychological perspective, altruistic behavior is an adaptive mechanism that increases chances of survival and reproduction for group-living species. The widely cited kin selection theory [15], which explains human altruistic behavior toward kin, suggests that even if one cannot directly pass on one’s genes to future generations, allowing those who share the same genes as oneself to survive and pass on their genes to future generations through altruism or sacrifice may be adaptive. An altruistic act of sacrificing oneself to help a kin is a loss to the individual but a gain from the perspective of the kin group, because the beneficiary is more likely to pass on their genes to future generations, which increases the number of people who share the same genes. An earlier study conducted by Burnstein et al. [16] lends support to this assumption of kin selection. Burnstein et al. presented participants with an array of vignettes that portrayed life-or-death or everyday situations involving triads of individuals. The participants were instructed to imagine that the three target individuals, who varied in genetic relatedness, were in need of help with the caveat that the participants possess sufficient time or resource to aid only one. The study found that the participants were more inclined to help their closest relative. Additionally, they found that the participants were more likely to opt for a kin in life-or-death situations compared with everyday situations [16].

Alternatively, altruistic behavior toward nonkin, which kin selection cannot explain, can be understood through theories anchored on the principle of reciprocity. From the perspective of indirect reciprocity for explaining prosocial behavior [17,18], altruism is viewed as a survival strategy developed by our ancestors while living in small groups. In essence, altruism toward ingroup members serves as a strategy for maintaining a favorable reputation in anticipation of a day when one may need help from ingroup members. This strategy is functional, because a good reputation within one’s group enhances the likelihood of receiving necessary help in the future [11,19].

Applying the abovementioned perspectives to the COVID-19 situation, we speculate that people may be more inclined to act altruistically toward the ingroup than the outgroup when feeling existentially threatened by COVID-19. Humans have lived in small, kin-based groups for the majority of history; thus, they have not evolved a perfect mechanism for distinguishing between kin and nonkin. Instead, they utilize physical similarities, attitudinal or cultural similarities, and geographic proximity as potential kinship cues [20]. Previous studies demonstrate that humans are more likely to help people who look like them. For example, when shown pictures of the faces of different children, including a morphing of their face, people perceive children with faces similar to them as attractive and were more likely to help these children, even if they were not genetically related [21]. People were also more likely to comply with the requests of an experimenter when they perceived that they share names, birthdays, or fingerprints with the experimenter than when they did not [22]. In an experiment utilizing an implicit association test, participants quickly associated persons with attitudes similar to theirs (e.g., thoughts about the death penalty) with word stimuli that indicated kinship such as *family*. Furthermore, in situations in which help is requested, people are more likely to help another person whose attitude is similar to theirs than a person whose attitude differs from theirs [23]. Taken together, these findings suggest that individuals use kinship cues, such as similarity, in nonkinship contexts to rapidly distinguish between ingroup and outgroup members. Moreover, they are more inclined to assist those perceived as kin. In light of these observations, the current study hypothesizes that amid the COVID-19 pandemic, individuals will be more likely to assist groups they consider related to them. The current study evaluates one’s intention to help a specific group on the basis of their willingness to distribute COVID-19 quarantine supplies. Consequently, we expect that individuals will exhibit greater readiness to dispense quarantine supplies to groups to which they identify.

### Variability in the definition of ingroup and outgroup

To test for ingroup bias, previous studies use the minimal group paradigm [24], which distinguishes ingroups from outgroups based on the gender or race of participants or use meaningless markers, such as colors or numbers, to prime group identity. The advantage of this approach is that it increases the internal validity of a study by controlling for exogenous variables that may be present in real-life situations. However, when using this method, process of identifying and evaluating the ingroup and outgroup may not reflect an individual’s feelings or attitudes toward a group, which results in a reduced external validity [25]. In addition, the definition of ingroup varies across individuals and cultures. For example, people in collectivistic cultures frequently identify themselves with stable groups, such as family and tribe, while people in individualistic cultures identify themselves with a larger and more diverse group of people, including not only family members but also coworkers and club members within the boundary of their ingroup [26]. Furthermore, the categories of groups with which one identifies and the definition of one’s ingroup can change across contexts [27,28]. However, to the best of our knowledge, studies that examine ingroup bias by accounting for individual and contextual variabilities in ingroup definition are lacking.

With the recognition that the definition of ingroup can vary among individuals, the current study proposed a novel measure for examining the extent to which people include diverse groups within their ingroup boundaries. Specifically, we assessed the *ingroup range* of individuals by asking participants to exclude those they did not consider part of their ingroup from a list of varied social groups. The elimination task [29], which is a common method used in the research on judgment and decision-making and employed by numerous psychological studies [e.g., 30, 31], inspired this approach. In line with the prior research on ingroup bias, the current study hypothesized that individuals with a broad ingroup range, as assessed using the abovementioned method, would be more likely to allocate resources to a more diverse set of social groups compared with those with a narrow ingroup range.

### Age differences in prosociality

Previous studies consistently demonstrated a relationship between age and prosociality. Scholars found that older participants are more likely to donate monetary reward from research participation to charity than their younger counterparts [32]. Furthermore, when asked to choose between receiving a reward and donating money to charity, older participants tended to favor donation over personal reward [33]. Research utilizing the dictator game to explore age-related tendencies in resource allocation also revealed that older participants exhibit more prosocial behaviors, such as distributing a larger share of their money to fictitious participants in the game, compared with younger participants [34,35]. Moreover, a meta-analysis of altruism studies found that older adults demonstrated stronger altruistic tendencies than younger adults even when controlling for demographic characteristics such as income and education [36].

Research on prosociality in the context of COVID-19 aligns with these earlier findings. When asked about volunteering or providing help during the COVID-19 pandemic, older participants reported having volunteered more than younger and middle-aged participants did. Furthermore, middle-aged and older participants were more likely to offer emotional and material support to others during the pandemic than did younger participants [37].

### Interplay between ingroup range and intention to help by age

Ingroup range is a new variable introduced in this study; thus, no research has examined the interaction between ingroup range and age and its influence on the intention to help in the context of COVID-19. However, the existing research suggests that the psychological resources of older adults buffer the psychological impact of disasters. This notion implies that older adults may be less prone to exhibit ingroup bias during crises, such as COVID-19, compared with younger adults.

First, older individuals may have more experience with disasters and, therefore, may be more likely to share resources with outgroup members in need even in situations that trigger ingroup bias. Altruism born of suffering [38,39] refers to the concept that negative past experiences trigger altruism toward others. Negative and painful experiences are generally considered to exert a negative impact on the life of an individual, but a possibility exists that these experiences can lead to compassion, empathy, or altruism toward others [38]. The intriguing aspect of altruism born of suffering is that it extends beyond mere empathy with those who share similar negative experiences. It also involves the display of altruistic behavior toward individuals suffering from experiences that differ from one’s own [39,40]. Empirical studies support this idea. For example, scholars demonstrate that people with more painful experiences exhibit more prosocial attitudes toward tsunami victims [41]. Furthermore, research finds that ingroup bias in helping victims mediates this relationship: the more painful the experiences of people, the less likely they believe that only ingroup victims should be helped. This reduction in ingroup bias leads to more prosocial attitudes toward tsunami victims as a whole. Inferring from these findings, another possibility is that older people, who have experienced relatively more life pain and trials than did younger people, are less likely to exhibit ingroup bias due to their extended altruism in the context of COVID-19.

In a similar vein, the notion that older people possess more psychological resources for coping with emergencies, such as natural disasters, which may lead to a broad distribution of resources, is possible. The elderly are frequently deemed a vulnerable group to natural disasters. However, several studies observe that older adults are actually less vulnerable to the psychological effects of disasters compared with younger survivors [42–44]. Examining mental health among earthquake survivors, studies find that older individuals exhibit higher levels of overall mental health than did younger individuals even after controlling for demographic characteristics such as marital status, gender, and employment status [44]. Even in the context of COVID-19, scholars report that older adults are less concerned about the threat posed by COVID-19 to several aspects of their lives, such as emotional well-being, financial situation, and work goals, compared with younger and middle-aged adults [45]. This result may be due to the fact that older people are more likely to engage in emotional optimization, which involves minimizing negative emotions while maximizing positive emotions in their daily lives, to achieve emotional satisfaction [46]. According to broaden-and-build theory, positive emotions, such as satisfaction, broaden the range of the thoughts and behaviors of people, while negative emotions, such as fear, narrow them [47,48]. Indeed, studies indicate that individuals experiencing positive emotions tend to identify a broader range of people as members of their ingroup [49,50]. Furthermore, when individuals are temporarily induced to experience positive emotions, they tend to engage in more helping behaviors [51]. In crisis situations, such as COVID-19, older individuals, who generally maintain relatively better mental health, may experience more positive emotions than their younger counterparts [43,52]. This tendency may predispose them to extend assistance to a diverse array of social groups. In contrast, younger individuals, who potentially perceive the threat of COVID-19 as greater due to its impact on their daily lives, may exhibit a relatively stronger ingroup bias.

### Current research

This study aimed to explore the relationship between the ingroup range of individuals and their intention to help different social groups and to assess if this relationship would vary with age. Specifically, we examined whether or not individuals with a broad ingroup range would display the intention to help a wider variety of social groups compared with those with a narrow ingroup range and whether or not age potentially moderates this relationship.

## Method

### Ethical considerations

The study received approval from the Institutional Review Board of Sungshin Women’s University (approval number: SSWUIRB-2020-032). Following this approval, an online survey was conducted from mid-September to early October 2020, immediately prior to the third wave of the COVID-19 pandemic when the mask mandate of the South Korean government was implemented.

Participants were informed about their rights, as well as the potential benefits and harms associated with participation, before the start of the study. A comprehensive research guide detailing the study, the rewards for participation, and the participants’ right to withdraw at any time was presented prior to the start of the online survey. This guide also included IRB information and contact details for the researchers to address any inquiries.

Participation in the study was voluntary, with only those who read the guide and agreed to participate joining the study. Informed consent was obtained by having participants read the study information sheet and check a box indicating their agreement.

The study adhered to the research ethics requirements set by the IRB. To ensure participant anonymity, no personally identifiable information, such as names or addresses, was collected.

### Participants

Using G*Power 3.1.9.7, we estimated a minimum sample size of 130 participants for the regression analysis, assuming an effect size of .10, a significance level of .05, and a power of .95. To account for potential dropouts due to the wide age range, we recruited over 300 participants to ensure sufficient power. A total of 349 South Koreans living in South Korea participated in the study online. We excluded data from one respondent who answered “no” to the informed consent question. Additionally, 42 individuals with missing data and 15 unreliable respondents (e.g., those who answered all questions using a single option) were excluded from the analysis, which left a final sample of 291 respondents (female: 225, male: 65, other: 1). The age of the respondents ranged from 19 to 62 years (*M* = 31.91, *SD* = 11.99). Most participants held a bachelor’s degree (*n* = 161, 55.3%), followed by those with a high school diploma (*n* = 79, 27.1%), a master’s degree (*n* = 28, 9.6%), an associate degree (*n* = 19, 6.5%), and a doctoral degree (*n* = 4, 1.4%). Given that the higher education attainment rate for South Koreans aged 25 to 64 was projected to reach 50.7% in 2020 [53], the fact that more than half of the participants hold a bachelor’s degree may reflect the broader educational characteristics of the Korean population. The materials and data file are available in the supporting information files.

### Measures

#### Ingroup range

The participants were presented with a list of social groups and asked to cross out any groups that they did not perceive as sharing the same characteristics as theirs. The list of groups was adapted from the social distance research by Park [54] and Bogardus [55], which consisted of the following: family, friends, coworkers and school alumni, neighbors, acquaintances, people from the same area, people from other areas, LGBTQ, North Korean defectors, refugees, migrants, Koreans living abroad, overseas Koreans of other nationalities, and nonKoreans. We calculated ingroup range as *1—the proportion of the groups that respondents eliminated* with high scores indicating wide ingroup ranges.

#### Intention to help

We asked the participants to imagine that they received a large amount of COVID-19 quarantine supplies, including face masks, protective clothing, and test kits. We then presented them with the same list of social groups as in the ingroup range task and asked them to select all groups to which they would like to distribute the supplies. In the analysis, we used the number of groups that the participants selected with high values indicating greater willingness to help a wide range of groups.

#### Age

We measured age as a moderating variable by asking the participants to enter their age as a number.

#### Demographic characteristics

The study measured the following demographic characteristics: gender, region of residence, religious affiliation, and socioeconomic status. For socioeconomic status, we measured level of education and average monthly household income to assess the objective socioeconomic status of the participants. Furthermore, we used McArthur’s ladder [56] to assess subjective socioeconomic status.

*Control variables*. Given the previous findings that trust is strongly associated with individual and intergroup cooperation [57], we measured as control variables the extent to which participants found society, the government, and media to be trustworthy using a single item for each (0: *not trustworthy at all*, 10: *very trustworthy*). In addition, based on research that demonstrate that age is closely related to the perceived likelihood of contracting COVID-19 and perceived COVID-19 threat of an individual [58–60], we measured perceived vulnerability to COVID-19 as a control variable. We used the perceived vulnerability to disease (PVD) questionnaire by Duncan et al. [61], which was translated and adapted for the COVID-19 context (1: *strongly disagree*, 6: *strongly agree*).

## Results

### Descriptive statistics and correlations

Following Kline [62]’s criteria, which define a violation of the assumption of normality when the absolute value of skewness surpasses 3 and the absolute value of kurtosis exceeds 10, all variables in this study met the normality assumption (Table 1). In the subsequent correlation analyses (Table 1), we found that age was positively correlated with the intention to help (*r* = .23, *p* < .001). Furthermore, we observed increases in educational attainment (*r* = .43, *p* < .001), subjective socioeconomic status (*r* = .17, *p* < .01), average monthly household income (*r* = .22, *p* < .001), and trust in society (*r* = .30, *p* < .001), the government (*r* = .21, *p* < .001), and media (*r* = .15, *p* < .01) with the increase in age. However, the perceived vulnerability to COVID-19 decreased (*r* = −.13, *p* < .05) with the increase in age. Moreover, a wider ingroup range was associated with a greater intention to help (*r* = .16, *p* < .01) and high levels of trust in society (*r* = .18, *p* < .01), the government (*r* = .19, *p* < .001), and media (*r* = .14, *p* < .05). Lastly, the study found that an increased intention to help was correlated with high subjective socioeconomic status (*r* = .12, *p* < .05), greater average monthly household income (*r* = .16, *p* < .01), and high levels of trust in society (*r* = .15, *p* < .05) and the government (*r* = .16, *p* < .01).

**Table 1:** Descriptive statistics and correlations among variables.

		1	2	3	4	5	6	7	8	9	10
1	Ingroup range	-									
2	Intention to help	.16**	-								
3	Age	.02	.23***	-							
4	Educational attainment	.03	.07	.43***	-						
5	Subjective socioeconomic status	.06	.12*	.17**	.18**	-					
6	Average monthly household income	.06	.16**	.22***	.15**	.32***	-				
7	Trust in society	.18**	.15*	.30***	.15*	.16**	.04	-			
8	Trust in government	.19***	.16**	.21***	−.05	.01	.06	.45***	-		
9	Trust in media	.14*	.09	.15**	.00	.08	.06	.44***	.59***	-	
10	Perceived COVID-19 vulnerability	.07	−.08	−.13*	−.11	−.15*	−.19**	−.02	.07	−.04	-
*M*		0.63	5.05	31.91	5.52	5.75	4.88	5.78	7.34	6.27	3.17
*SD*		0.23	3.74	11.99	1.04	1.58	3.07	2.06	2.49	2.35	0.50
Skewness		−0.34	1.45	1.15	−0.33	−0.15	0.06	−0.29	−0.55	−0.22	−0.08
Kurtosis		−1.11	1.05	-0.04	−0.84	−0.42	−1.03	−0.52	−0.38	−0.69	0.25

*Note*.

*p < .05;

**p < .01;

***p < .001.

### Effect of ingroup range on intention to help

To examine the relationship between ingroup range and intention to help, we conducted a hierarchical regression analysis. We entered subjective socioeconomic status, average monthly household income, and trust in society, the government, and media, which were significantly associated with ingroup range and intention to help, in step 1 and ingroup range in step 2.

Analysis demonstrated that the variables correlated with ingroup range and intention to help explained 5.7% of variation in the intention to help. Adding ingroup range in step 2 accounted for an additional 1.3% of variance in the intention to help, which increased explanatory power from 5.7% to 7%. Furthermore, ingroup range significantly predicted intention to help (*β* = .12, *p* < .05) even after controlling for variables that were correlated with ingroup range and intention to help. Table 2 indicates that a one-unit increase in ingroup range was associated with an increase of 1.87 in intention to help, that is, people with a wider ingroup range were more willing to distribute quarantine supplies to more diverse social groups.

**Table 2:** Hierarchical regression results for the relationship between ingroup range and intention to help.

Variables		Outcome Variable: Intention to Help						
		*B*	*SE*	*β*	*R* ^*2*^	*Adjusted R* ^*2*^	*ΔR* ^*2*^	*F*
1	Subjective socioeconomic status	0.15	0.15	0.06	0.06	0.04	0.06	3.47
	Average monthly household income	0.16	0.07	0.13*				
	Trust in society	0.16	0.12	0.09				
	Trust in government	0.20	0.11	0.14				
	Trust in media	−0.08	0.12	-0.05				
2	Subjective socioeconomic status	0.14	0.15	0.06	0.07	0.05	0.01	3.57
	Average monthly household income	0.15	0.07	0.12*				
	Trust in society	0.14	0.12	0.08				
	Trust in government	0.18	0.11	0.12				
	Trust in media	−0.08	0.12	−0.05				
	Ingroup range	1.87	0.95	0.12*				

*Note*.

*p < .05.

### Moderating effect of age

To examine whether or not the relationship between ingroup range and intention to help varied according to age, we used Process Macro model 1 by Hayes [63]. Analysis included ingroup range as the predictor variable, age as the moderator variable, and intention to help as the outcome variable. Ingroup range and age were mean-centered prior to analysis. We entered subjective socioeconomic status, average monthly household income, trust in society, the government, and media, and perceived vulnerability to COVID-19 as covariates.

Analysis revealed the significant main effects of ingroup range (*B* = 2.14, *p* < .05) and age (*B* = .06, *p* < .01) on intention to help; in other words, the participants with a wider ingroup range as well as older participants were more likely to display an intention to distribute quarantine supplies to more diverse social groups compared with their respective counterparts. Importantly, the moderating effect of age on the relationship between ingroup range and intention to help was significant (*B* = −.19, *p* < .05).

**Fig 1:**
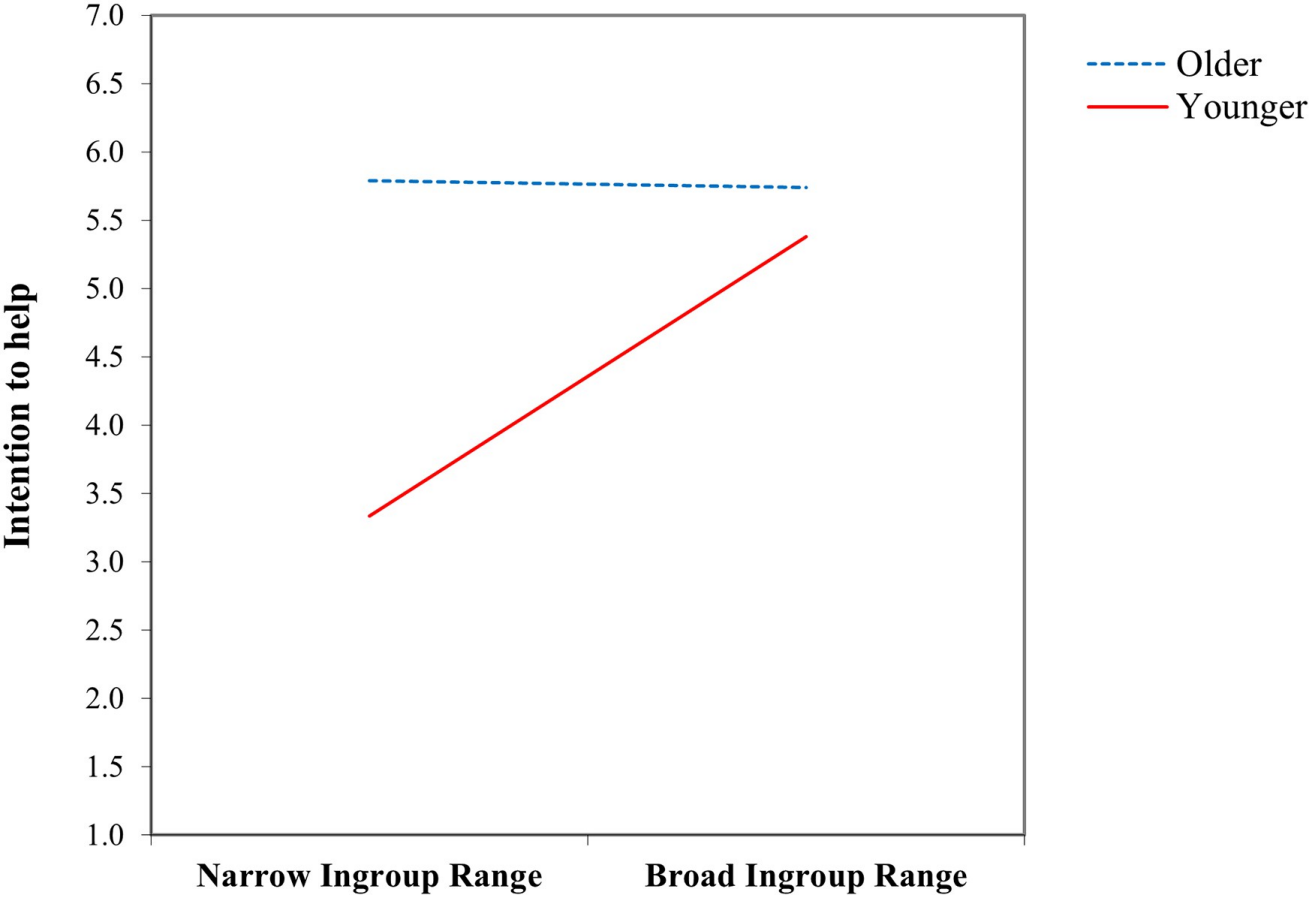
Moderating effect of age in the relationship between ingroup range and intention to help.

To investigate the aforementioned interaction, we specifically examined the conditional effects of age on the relationship between ingroup range and intention to help by applying the pick-a-point approach [64] at a level of ±1 SD from the mean age. We found that intention to help among relatively older participants did not vary in relation to ingroup range [*θ*_X → Y_|(*M* = 11.985) = −.11, *t*(281) = −.09, *p* = .932, *CI* = −2.64–2.42]. In contrast, for the younger participants [*θ*_X → Y_|(*M* = −11.985) = 4.40, *t*(281) = 3.32, *p* < .01, *CI* = 1.79–7.01] and those of average age [*θ*_X → Y_|(*M* = .000) = 2.14, *t*(281) = 2.31, *p* < .05, *CI* = .31–3.98], we observed a significant variation in intention to help as a function of ingroup range. This result suggested that the intention to help increases significantly with the increase in the ingroup range of participants who falls at or below the average age (Fig 1 and Table 3).

**Table 3:** Moderating effect of age in the relationship between ingroup range and intention to help.

	Coefficient (*B*)	*SE*	*t*
Intercept (Constant)	4.38	1.84	2.38*
Ingroup range (X)	2.14	0.93	2.30*
Age (M)	0.06	0.02	3.08**
X * M	−0.19	0.08	−2.46*
Subjective socioeconomic status	0.08	0.14	0.56
Average monthly household income	0.10	0.07	1.40
Trust in society	0.06	0.12	0.45
Trust in government	0.14	0.11	1.29
Trust in media	−0.05	0.11	−0.42
Perceived COVID-19 vulnerability	−0.43	0.44	−0.98

*Note*.

*p < .05;

**p < .01.

## Discussion

This study aimed to examine whether or not ingroup range would be related to the intention to help diverse social groups in the context of COVID-19 and to assess whether or not age moderates this relationship. We found that individuals with a broad ingroup range were more likely to display the intention to provide COVID-19 quarantine supplies to a wide set of social groups, and importantly that this tendency varied by age. Specifically, among older participants, intention to help did not vary by ingroup range, but among participants below or at the average age, those with a wide ingroup range intended to help more social groups.

The result that a wider ingroup range is associated with greater intention to help a wide range of social groups is consistent with that of previous literature on ingroup bias, which suggests that people allocate more resources to ingroup than outgroup members [4–6]. Additionally, the result implies that measuring the ingroup range unique to an individual, instead of arbitrarily distinguishing between ingroup and outgroup, yields results convergent with those on ingroup bias.

The study identified an intriguing pattern regarding the intention to help and ingroup range among different age groups. We noted that the intention to help of older participants did not vary depending on ingroup range. However, among participants below or at the average age, we observed an increase in intention to help with a broad ingroup range. This finding resonates with two possibilities presented in the Introduction. First, the observed moderating effect of age may be attributable to the propensity of older individuals to extend help to a more diverse range of social groups, which is an act of altruism that may be rooted in their experiences of hardship. This finding contrasts with the behavior of younger individuals who seem less inclined to do the same. The second possibility pertains to the emotional resilience of older individuals. A possible reason is that they possess superior emotion regulation skills and an abundance of psychological resources for managing disaster situations. This could make them more open to sharing quarantine supplies with social groups that extend beyond their immediate ingroup. Notably, however, the study did not directly test these two possibilities. Consequently, we suggest that future studies should incorporate direct measures of life experience and emotional regulation. The results could enrich the analysis of the relationship between ingroup range and intention to behave prosocially, which could provide a more nuanced understanding of the factors that motivate helping behavior across age groups.

The key contribution of the present study lies in the development of a novel measure for the distinct ingroup range of individuals. The study used this measure to revalidate the pervasive phenomenon of ingroup bias and to examine the factors that influence the manifestation of prosocial behavior amid the pandemic across age groups. The concept of an ingroup is fluid, that is, it shifts on the basis of cultural context [26] and groups with which individuals identify [27,28]. In today’s world, many societies form a rich tapestry of diversity that encompasses varied races, religions, values, and other contexts. As the sociocultural backgrounds of individuals become increasingly heterogeneous and commonalities decrease, the ingroup range of an individual is anticipated to be broader than ever. Moreover, the determinants that shape an individual’s definition of its ingroup are likely to be multifaceted. Therefore, understanding the factors utilized by individuals to define their ingroup and individual variances in ingroup range holds important implications. Such an understanding could provide valuable insight into the dynamics of interpersonal relationships, group interactions, and intergroup relations within multicultural societies. In essence, any future research that intends to explore the three abovementioned aspects would benefit from employing a measure similar to the one used in the current study. Such a measure could enhance the ecological validity of research by accurately reflecting the social context of the co-existence of diverse groups.

From a practical standpoint, the findings offer valuable insights that could be instrumental in designing interventions for potential future pandemics. Given that a wide ingroup range is positively linked to the intention to help only among younger participants, efforts to stimulate prosocial behaviors may seemingly be more beneficial if they are primarily targeted at younger people. An effective intervention may focus on equipping younger individuals with psychological resources necessary for managing disaster situations, which is similar to their older counterparts. For instance, we could consider initiating a program that encourages younger people to maintain gratitude journals on social media platforms and that offers rewards to those who consistently share gratitude entries over a specified time frame. In the context of the COVID-19 pandemic, research indicates that experiencing more gratitude can decrease perceived stress related to COVID-19 and enhance positive emotions [65]. As positive emotions are known to broaden the range of thoughts and behaviors [47,48], maintaining a gratitude journal can serve a dual purpose. It can not only help individuals build the necessary resources to address disaster threats but can also facilitate the expansion of their ingroup range. Thus, such a strategy may serve as a viable means of fostering prosociality during challenging times.

Despite the theoretical and practical implications discussed, this study is limited by its reliance on cross-sectional measurement of ingroup range and intention to help through a self-report questionnaire. Consequently, it was not possible to establish a clear causal relationship between the two variables or to determine if any observed association resulted from a direct influence. In other words, due to the limitations of the cross-sectional design, determining whether ingroup range acts as an antecedent of intention to help was unfeasible. To establish a clearer causal relationship between these variables, it would be necessary to examine whether intention to help changes in response to variations in ingroup range within a controlled experimental setting. Therefore, future research could manipulate ingroup range to assess its potential causal effect on the intention to help.

An experimental approach to exploring how ingroup range affects helping intentions could draw on the multi-layered social identity theory. This theory posits that individuals possess hierarchical identities that can merge ingroups and outgroups under shared categories [27,66,67]. Future research could thus manipulate ingroup range by introducing stimuli that prompt participants to include one of the social groups from the current study as an ingroup through higher-level common categorization. For instance, a narrow ingroup range could be induced by presenting categorization stimuli that apply only to a small number of groups, such as nationality, region of residence, or language spoken. Conversely, under a wide ingroup range condition, participants could be exposed to stimuli that evoke a sense of cosmopolitanism [68], fostering the perception of a global community across diverse nationalities, languages, and lifestyles. By manipulating ingroup range and examining whether participants assist groups they include in their ingroup, future studies could address the causal link between ingroup range and prosocial behavior, which the current study could not establish.

Finally, this study includes a small sample of Korean participants. Although the sample size met the minimum requirement for testing moderating effects, it may not have been large enough to robustly capture the effects we aimed to observe. Generally, a larger sample size reduces error and brings results closer to the true parameter, so recruiting more participants will be important in future research. Furthermore, generalizing these findings should be approached with caution. Most participants in this study held bachelor’s degrees, which, while reflecting the broader characteristics of the Korean population, raises uncertainty about whether the results would replicate in countries with differing educational demographics. Future studies should replicate these findings with participants more evenly distributed by education level, allowing us to better assess the generalizability of our findings beyond Korea.

## Conclusion

Taken together, this study devised a method for measuring ingroup range and demonstrated that the intention to help different social groups during the COVID-19 pandemic is dependent on one’s ingroup range and age. Researchers of infectious disease caution that another pandemic could break out at any time. We look forward to additional research on prosocial behavior during disasters, such as infectious disease outbreaks, to prepare for similar potential scenarios.

## Supporting information

S1 Dataset Dataset used in analysis. (XLSX)

S1 Materials Items belonging to ingroup range and helping intention. (PDF)
