# Supplementary material for: Moderating role of age in the relationship between ingroup range and intention to help during the COVID-19 pandemic
Source: PLoS One. 2025 Jan 24;20(1):e0316316. doi: 10.1371/journal.pone.0316316 (PMC11759371; doi:10.1371/journal.pone.0316316)
Supplement: S1 Materials — (PDF) [file pone.0316316.s002.pdf]

Materials

Ingroup Range

Below is a list of the different social groups that live with you. From the list below, please remove any group(s) that you do not feel you can identify with. There is no right or wrong answer to this question. So you can check all the group(s) you wish to remove.

(1) Family

(2) Friends

(3) Coworkers and school alumni

(4) Neighbors

(5) Acquaintances

(6) People from the same area

(7) People from other areas

(8) LGBTQ

(9) North Korean defectors

(10) Refugees

(11) Migrants

(12) Koreans living abroad

(13) Overseas Koreans of other nationalities

(14) NonKoreans

Intention to help

Imagine that one day, you have a large amount of COVID-19 quarantine supplies. You want to distribute the supplies, such as face masks, hand sanitizer, and test kits, to the people you want to give them to. Below is a list of social groups that you would like to receive your supplies. Which group(s) would you like to distribute quarantine supplies to? There is no right or wrong answer to this question, so please select all the group(s) you would like to distribute supplies to. Do not select any group(s) you do not want to distribute quarantine supplies to.

(1) Family

(2) Friends

(3) Coworkers and school alumni

(4) Neighbors

(5) Acquaintances

(6) People from the same area

(7) People from other areas

(8) LGBTQ

(9) North Korean defectors

(10) Refugees

(11) Migrants

(12) Koreans living abroad

(13) Overseas Koreans of other nationalities

(14) NonKoreans
